# Parenting as an inhibitor of gender disparities in alcohol use: the case of early adolescents in China

**DOI:** 10.1186/s12889-020-09195-2

**Published:** 2020-07-13

**Authors:** Ai Bo, James Jaccard

**Affiliations:** 1 Helen Bader School of Social Welfare, Department of Social Work, University of Wisconsin-Milwaukee, 2400 E. Hartford Ave, Milwaukee, WI 53201 USA; 2 School of Social Work, The University of North Carolina at Chapel Hill, 325 Pittsboro St., Campus Box 3550, Chapel Hill, NC 27599 USA; 3 Silver School of Social Work, New York University, 1 Washington Square North, New York, NY 10003 USA

**Keywords:** Parental monitoring, Parental empathy, Parental involvement, Underage drinking

## Abstract

**Background:**

Gender differences in alcohol use are more substantial among early adolescents in China than in the United States, presumably because of more permissive drinking norms for boys than girls in Chinese culture. This study tested a theory that gender differences in early experimentation with alcohol can be reduced through general parenting practices. Whereas traditional research has identified mediators of gender differences in alcohol use, the current research isolated moderators of gender differences and developed their implications for prevention programs.

**Methods:**

The study analyzed the data from the China Global School-Based Student Health Survey (*n* = 8805 middle school students in four cities). Youth completed anonymous surveys in classroom settings. The study examined interaction effects between gender and parenting variables using multiple regression with robust standard errors.

**Results:**

Early adolescent boys exhibited higher levels of drinking than girls for all drinking outcomes. The gender differences in drinking were negatively associated with the level of perceived parental monitoring, parental involvement in adolescent school performance, and parental empathy in a nonlinear way.

**Conclusions:**

Results suggested that early adolescents’ perceptions of general parenting practice nonlinearly moderated gender disparities in alcohol use.

## Background

Alcohol use is a leading risk factor contributing to the global disease burden in 10- to 24-year-olds (7% of disability-adjusted life-years) [1]. Early initiation of alcohol use (before age 14) is associated with serious health consequences including increased risk of future alcohol dependence and abuse [2], alcohol-related motor vehicle crashes [3], and other unintentional injuries [4]. Increased alcohol consumption in China has heavily contributed to recent increasing global trends of alcohol consumption per capita [5]. Alcohol consumption in China is projected to increase during the coming decade given rapid economic growth and weak alcohol deterrence policies (e.g., lack of enforcement of age restrictions) [5]. Adolescent drinking rates and related negative consequences have also been escalating in major Chinese cities [6, 7]. A study conducted in 2004 with 21,430 middle school (7th to 9th grade) students in 18 major Chinese cities found that 16% of adolescents reported past 30-day alcohol use [6]. A recent meta-analysis found a past-30-day alcohol use rate of 19% for Chinese middle schoolers [7].

Although alcohol use prevalence rates do not differ much by gender until youth reach young adulthood in the United States and most European countries [8–11], such gender differences are common in early experimentation with alcohol among Chinese adolescents; Chinese adolescent boys exhibit higher drinking prevalence than adolescent girls [6, 7]. Of interest is identifying variables that buffer or dampen these gender differences in early experimentation with alcohol, ideally with a focus on variables that are modifiable so that they can be addressed in prevention programs. No prior research has explored such buffers for Chinese youth. Understanding the bases of adolescent gender differences in alcohol use is important not only for alcohol prevention efforts in China, but also for addressing drinking by Chinese immigrants in Western countries (e.g., the United States) because immigrant families bring with them strong foundations in Chinese culture and traditions.

Scholars have offered explanations for the gender gap in adolescent drinking patterns and drinking progression when such differences occur, with one of the most prominent explanations being a “double standard” for drinking for boys and girls based on traditional gender roles and stereotypes [12, 13]. Greater drinking among boys is associated with stereotypical masculine qualities, which reinforce their drinking, whereas traditional feminine attributes are associated with less drinking [12, 14, 15]. The double standard of drinking for boys versus girls based on gender roles is prominent in the Chinese context: Chinese women are discouraged from drinking by Chinese cultural gender norms, leading to women perceiving more disadvantages of drinking than men [16].

Although identification of mediators of gender differences (e.g., gender roles) is important, it also is crucial to understand moderators of the relationship between gender and adolescent drinking, i.e., identifying contexts and situations where both genders tend to abstain from drinking compared to contexts where boys drink more than girls. The present research focuses on the family context. Parents play key roles in socializing adolescents. Relationships between parent variables (e.g., parental drinking behaviors, parent-child relationship quality, general parenting behaviors, alcohol-specific parenting behaviors) and adolescent drinking have been well documented [17]. Parenting behaviors can reinforce gender norms and gender-specific factors associated with experimentation with alcohol during early adolescence. However, the research findings are mixed on whether parenting factors have differential effects on youth drinking as a function of gender [13]. In some research, adolescents’ perceptions of parental monitoring and parental support tend to be associated with less alcohol use in both genders (although the magnitude of the effect may differ by gender) [18–20], whereas, other studies find that adolescent-reported or parent-reported parental monitoring is associated with less drinking among boys only [21, 22].

In contrast to these studies conducted on youth from Western countries, limited research has examined questions relating gendered drinking to the family context in Chinese youth. Alcohol-related research with Chinese youth has been largely epidemiological, emphasizing drinking patterns [23, 24] and documenting the relationship between alcohol use and other problem behaviors (e.g., smoking, sexual risk behaviors) [6, 25]. Only a few studies have reported associations between parental factors and adolescent drinking. A study with 1183 6th grade Taiwan students found that alcohol initiation was associated with having parents who both used alcohol, provided less parental support, and had more family conflict [26]. However, this study did not explore gender differences. Another study with 777 10th graders in Taiwan found that the influence of parental drinking on adolescent drinking is more pronounced among offspring of the same sex [27]. A qualitative study of 101 Hong Kong Chinese youth between the ages of 15 and 19 found that girls as compared to boys were more concerned about their parents’ negative view of teenage drinking and therefore did not drink in order not to disappoint their parents [28]. Overall, previous studies have not investigated modifiable factors that can address gender disparities in early experimentation with alcohol among Chinese adolescents. The present research addresses this knowledge gap.

### The present study

This study investigated three modifiable parenting dimensions—parental monitoring, parental involvement in adolescent school performance, and parental empathy—as possible moderators of gender differences in alcohol use. The study focused on adolescent perceptions of these constructs rather than parental behavior per se because there is considerable research to indicate that adolescent reports of parenting behavior are far more predictive of youth problem behaviors than parent reports of parenting behavior [29–31]. Constructivist theories argue that how people construe their world is central to determining the decisions they make and the behaviors they engage in [32]. This is likely true for adolescent construals of parental monitoring, parental involvement, and parental empathy as well. We hypothesize that higher levels of perceived parental monitoring, perceived parental empathy, and perceived parental involvement in adolescent school performance are associated with smaller gender differences in adolescent drinking. We hypothesize that each parenting dimension has a distinct underlying mechanism for moderating gender differences in Chinese adolescent drinking, which we now consider, in turn.

#### Parental monitoring

Adolescents’ perceived parental monitoring is negatively associated with their drinking behaviors in general [18–20]. In our study, we hypothesize that perceived parental monitoring should be associated with less drinking for both girls and boys due to more limited opportunities for them to engage in drinking as a result of parental supervision. However, we also hypothesize that increased parental monitoring will lessen boys’ perceptions that it is permissible for boys to drink (a Chinese cultural norm) due to increased parental vigilance, whereas girls already perceive drinking is unacceptable for them. Thus, high levels of parental monitoring will associate with smaller gender differences in drinking.

#### Parental empathy

Parental empathy can maintain a sense of connection with parents, and alleviate stress, which in turn can serve as a protective factor for adolescent drinking [18]. In China, families have long valued sons over daughters and tend to give sons preferential treatment, which may enhance sons’ emotional responses to parental empathy [33, 34]. In addition, girls generally receive more emotional support from friends than boys [35]. Thus, when perceived parental understanding is lacking, boys are more likely to have difficulties coping with their stress than girls, and show increased drinking, whereas girls’ drinking should be relatively constant across attributions of parental empathy. Thus, a family context characterized by low levels of parental empathy should be associated with larger gender differences in alcohol experimentation, whereas a family context characterized by high levels of parental empathy should be associated with more modest differences.

#### Parental involvement in adolescent school performance

Research suggests that parental involvement with their children tends to buffer against the effect of mental distress on alcohol use [36]. For perceived parental involvement in adolescent school performance, if adolescents perceive their parents as involved and supportive in their school success, they might feel less stressed given broader societal pressures to do well academically. Recent research suggests Chinese girls have more non-familial support networks for performing well in school than boys, such as more encouragement from teachers and more same-sex peer role models who value school [37, 38]. When parents are less involved, girls have these other sources of support to rely on but this is less true of boys. As a result, lack of parental involvement with school performance is likely to be more stressful and impactful for boys, which is associated with increased risk of experimentation with alcohol. Thus, a family context characterized by low levels of parental involvement in school performance is likely associated with larger gender differences in alcohol experimentation than a family context with high levels of parental involvement in school performance.

In sum, the current research explored gender differences in early alcohol experimentation and consumption among Chinese adolescents. It explored how family contexts can potentially enhance or diminish such gender differences, focusing on three modifiable parenting constructs, namely adolescents’ perceived parental monitoring, parental empathy, and parental involvement in school performance. The research documents gender differences in adolescent alcohol consumption as a function of family contexts and has potential implications for the design of parent-based prevention programs both for youth in China and Chinese immigrant youth overseas.

## Methods

### Respondents

This study involved secondary data analysis of the China Global School-Based Student Health Survey (GSHS) [39, 40]. We analyzed the data from 8805 middle school students (Grades 7 to 9) in four cities in China [40]. Because these are public, de-identified data sets, the research was classified as exempt by the New York University Institutional Review Board.

### Procedure

China GSHS used a two-stage cluster sampling approach [41]. During the first stage, 25 schools were selected from each of four cities—Beijing, Hangzhou, Wuhan, and Urumchi—using probability sampling from all middle schools in the cities. Intact classrooms were then randomly selected in the second stage from each school of the 100 selected schools. Students in each classroom participated in anonymous surveys during one class period by completing a self-administered questionnaire and recorded their responses on a computer scannable answer sheet. China GSHS had a school response rate of 100%, and a student response rate of 98% [41]. The data were collected in 2003.

### Measures

Questions were developed based on the Youth Risk Behavior Survey questionnaire, which has been used to assess priority health-risk behaviors among 9th- to 12th-grade students in the United States biennially since 1991 [42]. Youth Risk Behavior Survey and GSHS questionnaires have been pilot tested and adapted widely in assessing health-risk behaviors in mainland China, with empirically documented acceptable levels of reliability and validity [6].

#### Alcohol use

Alcohol use was measured by four items focused on the frequency of alcohol use, the amount of alcohol use, episodes of heavy drinking, and problems associated with alcohol use. Frequency of alcohol use was measured as the “number of days you had at least one drink containing alcohol during the past 30 days” using a 7-point scale from “0 days,” “1 or 2 days,” “3 to 5 days,” “6 to 9 days,” “10 to 19 days,” “20 to 29 days,” and “All 30 days.” Amount of alcohol use was measured as the “number of drinks you usually drink per day on the days you drank alcohol during the past 30 days” using a 7-point scale from “did not drink,” “less than one drink,” “1 drink,” “2 drinks,” “3 drinks,” “4 drinks,” and “5 or more drinks.” Episodes of heavy drinking were defined as “the number of times you drank so much alcohol you got really drunk,” scored on a 4-point scale from “0 times,” “1 or 2 times,” “3 to 9 times,” “10 or more times.” Problems associated with alcohol use were measured as “the number of times you ever had a hangover, felt sick, got into trouble with your family or friends, missed school, or got into fights as a result of drinking alcohol” on the same metric with that of heavy drinking. All the values were recoded to represent the midpoints of each category (e.g., 1.5 = “1 or 2 days/times”).

Self-reports of alcohol use, of course, are subject to bias. The fact that the data were collected under conditions of anonymity and did not require face-to-face reporting of alcohol use increases their likely validity [43]. The validity and reliability of self-reported measures like those used in this research has empirical support [44, 45]. Del Boca and Darkes [46] conducted an extensive review of self-reports of alcohol use and concluded that “self-report methods offer a reliable and valid approach to measuring alcohol consumption.”

#### Perceptions of parenting

The China GSHS had three questions on protective parenting, requiring students to choose from categories on a 5-point metric (0 = *never*, 1 = *rarely*, 2 = *sometimes*, 3 = *most of the time*, and 4 = *always*). Perceived parental monitoring was measured by asking: “During the past 30 days, how often did your parents or guardians really know what you were doing with your free time?”; perceived parental empathy was measured by asking: “During the past 30 days, how often did your parents or guardians understand your problems and worries?”; and parental involvement in adolescent school performance was measured by asking: “During the past 30 days, how often did your parents or guardians check to see if your homework was done?” Although criticisms of single-item measures are common, a surprisingly large number of studies have found that multiple-item scales of a construct often predict no better than single-item representations of the same construct [47–49]. In the current case, the items have face validity and as will be shown, also have concurrent validity in that they predicted drinking in meaningful, predictable ways.

### Analytic methods

Analyses primarily explored interaction effects between gender and a given parenting behavior using linear multiple regression analyses [50]. Because outcomes were skewed, we used Huber-White based robust maximum likelihood estimation instead of traditional ordinary least squares standard errors. Missing data were negligible. Both weighted and unweighted analyses were conducted for all models, and the results were comparable in both cases. We report unweighted analyses given the non-informativeness of the sampling weights [51]. Adjustments for clustering at the school level were made in all analyses. Because preliminary analyses suggested complex nonlinear relationships between parenting variables and alcohol use outcomes, it was inappropriate to treat parenting measures as continuous and to model bilinear interactions (per traditional product terms) between parenting and gender. Parenting measures were therefore treated as ordinal and were represented by multiple dummy variables for each level of the parenting predictor. The coefficient for any given product term represents a single degree of freedom interaction contrast for the equivalent of a 2X2 factorial table that treats gender (male vs. female) as the focal independent variable and the two parenting levels (i.e., parenting level scored 1 and the parenting reference group) as the moderator variable. The significance test of the product term is a test of this interaction contrast [50]. These analyses allow us to estimate the average drinking outcomes for both male and female adolescents and gender differences at each level of parenting, and to compare gender differences in drinking outcomes for any two levels of the parenting measures. For a given parenting construct (e.g., monitoring), we statistically held constant the other mean-centered dummy parenting variables and mean-centered grade and city to yield analysis of covariance-like adjusted means. We conducted sensitivity analyses for two samples, as appropriate, one using the total sample and the other focusing only on current drinkers (i.e., had at least one drink containing alcohol during the past 30 days), eliminating non-drinkers.

## Results

### Initial analysis

Preliminary analyses tested if results needed to be qualified by grade and city by testing three-way interactions between the parenting variables, gender, and either city or grade. The three-way interactions generally were not statistically significant after controlling for family-wise error rates using a Holm-modified Bonferroni procedure; all were trivial in magnitude. The correlations between the perceived parenting behaviors were low to moderate (i.e., between .3 and .5). As such, collinearity was not an issue and the three parenting perceptions had considerable unique variance that merited treating them separately.

Table 1 and Table 2 present descriptive statistics stratified by gender. All drinking outcomes were statistically significantly (*p* < .05) higher in male than female adolescents for both the entire sample and current drinkers. For the parenting behaviors, the modal response for perceived parental monitoring was “most of the time” (25.2% response rate); for parental involvement in adolescent school performance it was “sometimes”; for parental empathy it was “rarely” (24.3%). Gender differences in perceived parental monitoring and parental empathy were negligible. Male adolescents had higher levels of perceived parental involvement than female adolescents.

**Table 1:** Characteristics of Study Participants: Demographics

	Total (*n* = 8805)	Males (*n* = 4276)	Females (*n* = 4471)
Age* (mean, SD)	13.72 (1.08)	13.79 (1.09)	13.65 (1.06)
Gender (% female)	51.1%	n/a	n/a
Grade			
7	30.7%	31.4%	29.6%
8	33.4%	33.2%	33.8%
9	35.9%	35.4%	36.7%
City/Site			
Beijing	26.5%	26.3%	26.9%
Hangzhou	20.3%	20.9%	19.9%
Wuhan	20.4%	19.9%	20.4%
Urumchi	32.7%	32.9%	32.8%

* Gender difference is statistically significant at *p* < .05; SD = standard deviation

**Table 2:** Characteristics of Study Participants: Perceived Parenting Behaviors and Drinking Outcomes

		Total (*n* = 8805)	Males (*n* = 4276)	Females (*n* = 4471)
Parental Monitoring (0–4)	Never	12.8%	13.0%	12.7%
	Rarely	21.8%	21.8%	21.9%
	Sometimes	20.1%	21.2%	19.0%
	Most of the time	25.3%	24.4%	26.1%
	Always	20.0%	19.7%	20.4%
Parental Involvement* (0–4)	Never	17.9%	15.4%	20.3%
	Rarely	18.4%	16.6%	20.2%
	Sometimes	26.0%	25.6%	26.4%
	Most of the time	19.4%	21.0%	17.9%
	Always	18.3%	21.4%	15.3%
Parental Empathy (0–4)	Never	17.8%	18.4%	17.2%
	Rarely	24.3%	24.3%	24.3%
	Sometimes	21.9%	22.0%	21.9%
	Most of the time	22.3%	21.8%	22.8%
	Always	13.7%	13.6%	13.8%
Drinking Frequency* (0–30 days) (mean, SD)		.67 (3.04)	.94 (3.66)	.41 (2.32)
	0 days	86.0%	81.8%	89.9%
	1 or 2 days	9.1%	11.0%	7.3%
	3 to 5 days	2.1%	3.1%	1.2%
	6 to 9 days	1.0%	1.4%	0.6%
	10 days and more	1.8%	2.6%	1.1%
Drinking Amount* (0–5 drinks) (mean, SD)		.13 (.41)	.18 (.50)	.09 (.30)
	0 drinks	84.5%	80.0%	88.7%
	Less than one drink	9.7%	11.8%	7.8%
	1 drink	4.3%	6.1%	2.8%
	2 drinks and more	1.5%	2.2%	.8%
Heavy Drinking* (0–10 times) (mean, SD)		.24 (.99)	.37 (1.27)	.12 (.60)
	0 times	89.7%	85.7%	93.5%
	1 or 2 times	8.8%	11.7%	6.0%
	3 times and more	1.5%	2.6%	0.4%
Drinking Problems* (0–10 times) (mean, SD)		.13 (.75)	.18 (.94)	.07 (.49)
	0 times	94.8%	93.0%	96.4%
	1 or 2 times	4.5%	5.7%	3.4%
	3 times and more	0.8%	1.3%	0.3%

* Gender difference is statistically significant at *p* < .05 for both the full sample and current drinkers; *SD* Standard deviation

### Main analysis

**Fig. 1:**
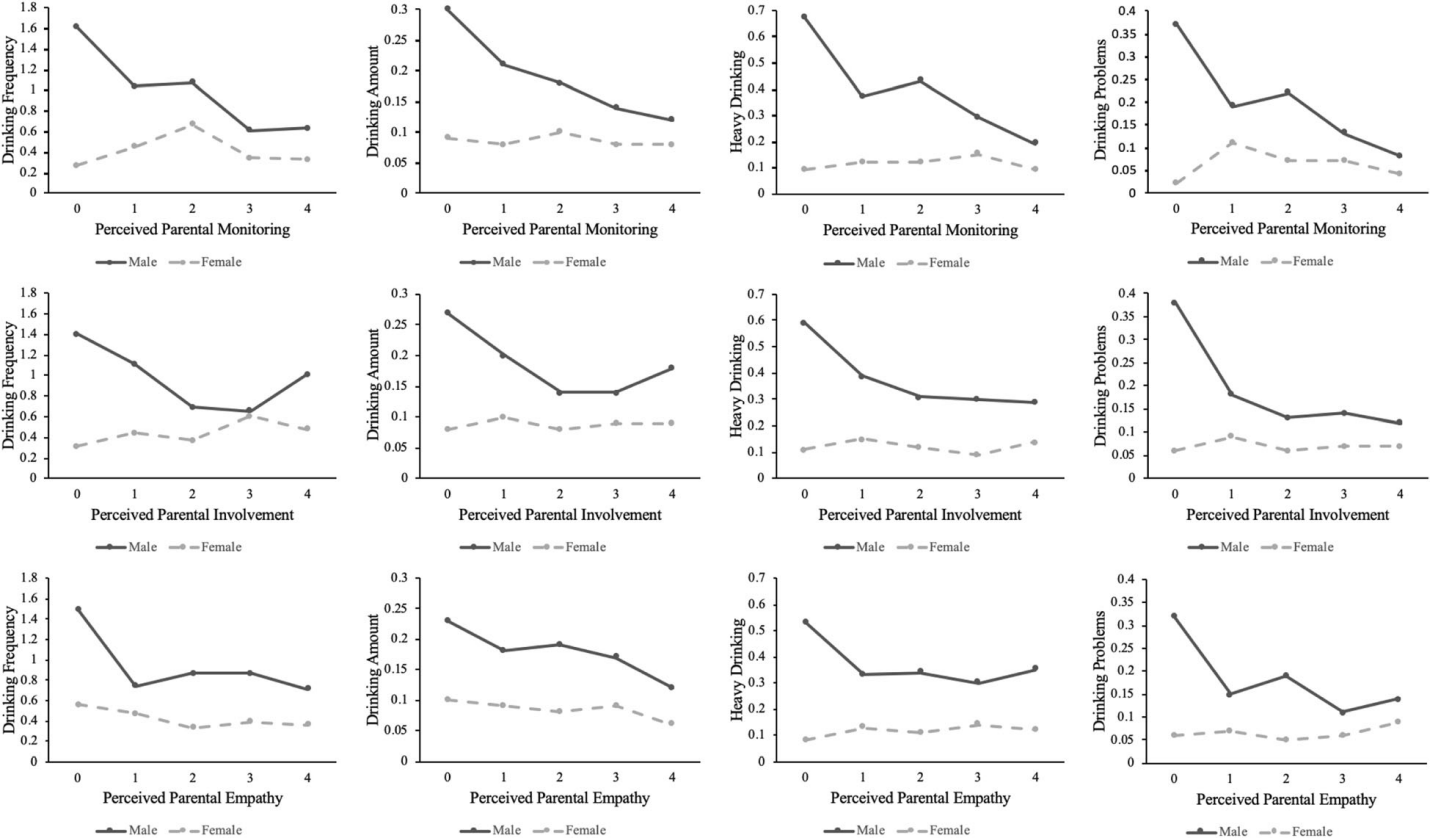
The Predicted Means of Drinking Outcomes as a Function of the Parenting Variables

Table 3 presents the predicted mean drinking outcome for each level of the perceived parenting variable as a function of gender for the full sample. Figure 1 presents the predicted means of drinking outcomes graphically as a function of the parenting variables for the full sample. There are several noteworthy trends. First, across all four drinking outcomes, there was a statistically significant gender difference in each alcohol outcome at every level of perceived parenting behavior for each parenting attribution (except for drinking frequency when the level of parental involvement equaled “most of the time”). Thus, the tendency for early adolescent Chinese boys to consume alcohol more than girls was pervasive. Having said that, the significance patterns are affected by the large sample size, so one also must consider the magnitude of coefficients, not just statistical significance. With this in mind, Fig. 1 shows a substantial separation between boys and girls when perceptions of parenting behavior were scored 0, signifying a “never” response to a given parenting question. The separation tended to diminish substantially once adolescent perceptions moved to the second response category (“rarely”), indicating that even some small degree of perceived positive parenting might influence gender differences in drinking relative to the “never” group. For most drinking outcomes and parenting behaviors, the change in gender drinking differences when perceptions of parenting shifted from “never” to “rarely” was statistically significant (*p* < .05).

**Table 3:** Gender as Predictors of Mean Drinking Outcomes by Each Level of Parental Perceptions (Full Sample)

	Drinking Frequency (*n* = 7924)			Drinking Amount (*n* = 8019)			Heavy Drinking (*n* = 8446)			Drinking Problems (*n* = 8494)		
	Male	Female	M-F Difference	Male	Female	M-F Difference	Male	Female	M-F Difference	Male	Female	M-F Difference
Parental Monitoring												
Never	**1.61**	0.27	1.34*	0.30	0.09	0.21*	**0.67**	0.09	0.58*	**0.37**	**0.02**	0.35*
Rarely	1.04	0.45	0.59*	0.21	0.08	0.13*	0.37	0.12	0.25*	0.19	0.11	0.08*
Sometimes	**1.07**	**0.67**	0.40*	**0.18**	0.10	0.08*	**0.43**	0.12	0.31*	**0.22**	0.07	0.15*
Most of the time	0.61	0.34	0.27*	0.14	0.08	0.06*	**0.29**	**0.15**	0.14*	0.13	0.07	0.06*
Always	0.63	0.33	0.30*	0.12	0.08	0.04*	0.19	0.09	0.10*	0.08	0.04	0.04*
Parental Involvement												
Never	1.40	0.31	1.09*	**0.27**	0.08	0.19*	**0.59**	0.11	0.48*	**0.38**	0.06	0.32*
Rarely	1.11	0.44	0.67*	**0.20**	0.10	0.10*	0.39	0.15	0.24*	0.18	0.09	0.09*
Sometimes	0.69	0.37	0.32*	0.14	0.08	0.06*	0.31	0.12	0.19*	0.13	0.06	0.07*
Most of the time	**0.65**	0.60	**0.05**	0.14	0.09	0.05*	0.30	0.09	0.21*	0.14	0.07	0.07*
Always	1.01	0.48	0.53*	0.18	0.09	0.09*	0.29	0.14	0.15*	0.12	0.07	0.05*
Parental Empathy												
Never	**1.50**	0.56	0.94*	0.23	0.10	0.13*	**0.53**	0.08	0.45*	**0.32**	0.06	0.26*
Rarely	0.74	0.47	0.27*	0.18	0.09	0.09*	0.33	0.13	0.20*	0.15	0.07	0.08*
Sometimes	0.87	0.33	0.54*	0.19	0.08	0.11*	0.34	0.11	0.23*	**0.19**	0.05	0.14*
Most of the time	0.87	0.39	0.48*	**0.17**	**0.09**	0.08*	0.30	0.14	0.16*	0.11	0.06	0.05*
Always	0.71	0.36	0.35*	0.12	0.06	0.06*	0.35	0.12	0.23*	0.14	0.09	0.05*

* Gender difference is statistically significant at *p* < .05; Bold numbers indicate statistically significant difference of current level from estimate at the next higher level; All estimates are adjusted for grade, site, and clustering; *M-F* Male minus Female

A second noteworthy trend in Fig. 1 (and corroborated in Table 3) is the relatively flat trendline in adolescent girls’ drinking as each facet of perceived positive parenting increased. This can be contrasted with the downward (but uneven) trajectory of drinking across the parenting facets for boys. The reductions of gender differences thus were primarily due to reduced drinking for boys as perceived positive parenting increased. For the most part, increases in perceived positive parenting were not associated with decreases in female drinking outcomes. There was an exception to the general pattern, where gender difference in drinking frequency significantly increased as parental involvement shifted from the “most of the time” category to the “always” category, primarily due to increased drinking outcomes for boys.

Table 4 presents the predicted mean drinking outcome for each level of the perceived parenting variable as a function of gender but focusing only on current drinkers. The most notable difference from the analysis of the full sample was that the statistically significant gender differences in drinking outcomes tended to be observed primarily at low levels of perceived parenting (e.g., “never”) rather than at every level of perceived parenting. The gender differences tended to fully dissipate at higher levels of perceived positive parenting behaviors. Most other results replicated the full sample analyses (e.g., significant changes in gender differences in drinking when perceptions of parenting shifted from “never” to “rarely”; almost no significant changes in adolescent girls’ drinking as each facet of perceived parenting increased).

**Table 4:** Gender as Predictors of Mean Drinking Outcomes by Each Level of Parental Perceptions (Current Drinker Only)

	Drinking Frequency (*n* = 1107)			Drinking Amount (*n* = 1287)			Heavy Drinking (*n* = 1682)			Drinking Problems (*n* = 1692)		
	Male	Female	M-F Difference	Male	Female	M-F Difference	Male	Female	M-F Difference	Male	Female	M-F Difference
Parental Monitoring												
Never	**6.52**	3.11	3.41*	**1.07**	0.73	0.34*	**1.54**	0.33	1.21*	**0.85**	**0.09**	0.76*
Rarely	4.73	4.07	0.66	0.84	0.65	0.19*	0.87	0.43	0.44*	0.46	0.37	0.09
Sometimes	**5.25**	5.59	−0.34	0.80	0.72	0.08	**1.00**	0.31	0.69*	**0.58**	0.23	0.35*
Most of the time	3.65	3.71	−0.06	0.72	0.70	0.02	0.68	0.41	0.27	0.30	0.12	0.18
Always	5.68	3.38	2.30	0.88	0.72	0.16	0.59	0.35	0.24	0.19	0.08	0.11
Parental Involvement												
Never	5.25	3.13	2.12*	**0.99**	0.70	0.29*	**1.44**	0.24	1.20*	**0.89**	0.18	0.71*
Rarely	4.75	3.71	1.04	0.78	0.70	0.08	0.81	0.40	0.41*	0.37	0.21	0.16
Sometimes	4.37	**3.50**	0.87	0.77	0.65	0.12	0.66	0.41	0.25	0.27	0.15	0.12
Most of the time	4.46	6.31	**−1.85**	0.81	0.76	0.05	0.96	0.28	0.68*	0.51	0.28	0.23
Always	5.85	3.90	1.95	0.88	0.67	0.21	0.73	0.60	0.13	0.29	0.14	0.15
Parental Empathy												
Never	**6.65**	4.52	2.13*	0.94	0.75	0.19*	**1.21**	0.21	1.00*	**0.77**	0.10	0.67*
Rarely	3.77	4.12	−0.35	0.84	0.71	0.13*	0.69	0.37	0.32*	0.33	0.13	0.20*
Sometimes	4.36	3.63	0.73	0.86	0.72	0.14	0.93	0.29	0.64*	0.45	0.18	0.27*
Most of the time	5.29	3.81	1.48	0.83	**0.72**	0.11	0.82	0.57	0.25	0.30	0.22	0.08
Always	5.37	3.88	1.49	0.71	0.46	0.25*	0.97	0.32	0.65*	0.43	0.47	−0.04

* Gender difference is statistically significant at *p* < .05; Bold numbers indicate statistically significant difference of current level from estimate at the next higher level; All estimates are adjusted for grade, site, and clustering; *M-F* Male minus Female

## Discussion

The present research replicated prior studies that have found gender differences in drinking by Chinese youth but in a more comprehensive way by considering distinct drinking outcomes and using large representative samples of urban youth. We found that early adolescent Chinese boys tended to drink more frequently, consume more when they drank, have more episodes of heavy drinking, and experience more problems associated with drinking compared to early adolescent Chinese girls. The consistency of the gender differences across outcomes is striking.

We evaluated the boundary conditions of the gender differences under the general hypothesis that they would be negatively associated with the three facets of positive parenting, namely adolescent construals of parental monitoring, parental involvement in adolescent school performance, and parental empathy. The logic models underlying our hypotheses were grounded in adolescent perceptions about parenting behavior rather than parenting behavior per se. Adolescent perceptions are likely influenced by actual parenting behavior, but research suggests it is adolescent construals of parenting behavior that matters most [29–31]. The results were generally consistent with these hypotheses, but had interesting subtleties. Among adolescents who believe their parents do not care about their whereabouts, understand their problems, or check their homework at all, gender differences in drinking were generally the largest. Even among adolescents who perceived their parents as minimally involved in their lives, gender differences in drinking were much smaller mainly due to lower levels of boys’ drinking. Gender differences in drinking were generally similar among adolescents with moderate and high levels of perceived positive parenting (i.e., represented by responses in the “sometimes,” “most of the time,” and “always” categories). This is consistent with previous research in the United States that finds that adolescents with the lowest level of perceived parental involvement are at higher risk for using alcohol than adolescents who perceived their parents as somewhat or highly involved in their lives [36]. Clearly, what seems to matter most is that adolescents believe their parents are not completely ignoring them on the three parenting facets we studied. Surprisingly, gender differences in drinking frequency were larger among adolescents who perceived the highest level of parental involvement than adolescents who perceived the second highest level of parental involvement. Perhaps the frequent parent involvement in school performance became intrusive and a source of stress for some boys at the highest level of involvement. Among current drinkers, gender differences in drinking outcomes were mostly statistically non-significant at higher levels of perceived positive parenting behaviors.

An interesting puzzle that emerged in the study was the relative lack of association between drinking dynamics and the different levels of attributions of positive parenting for adolescent girls. For the most part, the levels of perceived positive parenting were not associated with female drinking, only male drinking. Although this cannot be entirely attributed to matters of base rates, floor effects undoubtedly come into play given the relatively low base rates for girls shown in Table 2. However, even among current drinkers, drinking among adolescent girls was not associated significantly with levels of positive parenting. It is likely that drinking by adolescent girls is sufficiently discouraged by strong norms against female drinking, such as ‘girls should not drink’ or ‘most girls like me do not drink’ in both a cultural and family context in China, whereas for boys, the norms are less oppressive. Boys generally experience less cultural and family pressures to not drink and may even be encouraged to drink on certain occasions (e.g., celebrations), leading boys to engage in greater levels of experimentation with drinking. Girls who drink at such a young age (in middle school) may experience a multitude of factors encouraging their drinking (e.g., affiliating with deviant peers, traumatic experiences, depression) that make responses to drinking more recalcitrant to changes in positive parenting [52]. To be sure, drinking on the part of boys is also affected by such factors, but based on past research, girls may be more vulnerable to these factors and less influenced by parenting as a result [52]. Another potential reason for why girls are less responsive to parental involvement and parental empathy compared to boys could be that girls receive more support from friends and school [35, 38]. More fine-grained analyses of why girls’ drinking is less responsive to general parenting behaviors are needed.

Another important finding of the current research is that each of the parenting dimensions moderated gender differences in its own right, independent of the other parenting variables. The intercorrelations among the three parenting variables were modest and the fact that each was implicated in drinking dynamics while holding the others constant suggests each makes a unique contribution. Most research on these dimensions has focused on them in isolation rather than in a multivariate parenting context. It was useful to learn that even when considered in multivariate analyses, each of the dimensions contributed unique explained variance in its own right.

For parental monitoring, adolescents’ perceived threat of being caught in a transgression as a result of presumed parental monitoring likely outweighed the social norms that boys’ drinking is more acceptable than girls’ drinking, thereby homogenizing gender differences in drinking; boys showed lower levels of alcohol use with higher levels of parental monitoring, eventually exhibiting similar drinking patterns as girls. For parental empathy, higher levels of felt empathy and understanding likely increase the likelihood that adolescents will seek help and support from their parents when they experience stress and difficulties. To the extent that drinking is perceived as a coping strategy for stress [53], the intention to drink should decrease as adolescents perceive their parents will understand their problems and help them deal with them constructively. Given that Chinese boys are more responsive to parental empathy than girls [34, 35], boys’ drinking should be affected more by perceived parental empathy than girls’ drinking. For perceived parental involvement in adolescent school performance, given that boys have more limited support networks for performing well in school than girls (e.g., having less encouragement from teachers, poorer classroom behaviors, and fewer peer role model) [37, 38], lack of parental involvement in school work is likely more stressful for boys, which in turn could lead to higher levels of drinking for boys than girls. More research studying the unique mechanisms of each modifiable parenting factor on adolescent drinking outcomes is needed.

Theoretically, the present study represents a significant contribution to research traditions that seek to understand gender differences in adolescent risk behaviors more generally and alcohol use in particular. By focusing on moderators rather than mediators of gendered drinking, the research elucidated how the family context can potentially constrain the impact of gendered mediators (e.g., gender roles, cultural norms that encourage boys as opposed to girls to drink) and helped establish boundary conditions on those mediators. The research should thus foster more comprehensive analyses of gender dynamics that encourage the incorporation of both mediators and moderators into theoretical explanations of gender differences.

From a prevention perspective, the present research suggests that prevention programs addressing parental monitoring, parental empathy and support, and parental involvement with school performance may be influential for adolescent Chinese boys but less so for adolescent girls. Perhaps addressing these family variables will further reinforce low drinking rates for Chinese girls, but in terms of changing alcohol use, the potential effects of the perceived parenting variables seem limited to Chinese boys. Despite this, meta-analytic results suggest that parent-based programs aimed at increasing general and/or alcohol-specific parenting skills can prevent or reduce alcohol use among both boys and girls in Western countries including Asian American girls [54].

Intervention research on parent-based interventions in the United States support the malleable character of parenting behaviors [55–58]. A study of a sample of U.S. early adolescent girls found that parent-based interventions had effects on both parent-reported and adolescent-reported parenting (e.g., parental monitoring, empathy) and adolescent drinking [55]. Several studies also found significant mediational roles for parent-reports of parenting [56] and adolescent-reports of parenting [57, 58] for parenting programs focused on adolescent drinking. Unfortunately, the effects and mechanisms of parenting programs for preventing Chinese adolescent drinking are unstudied. More formal intervention research is needed to evaluate the efficacy, effectiveness, and mechanisms of parent-based interventions for Chinese adolescent drinking.

The data we analyzed were collected in 2003, which raises the possibility that gender differences in drinking may have changed since then. However, more current data suggests that the gender difference in drinking still exists in China [7]. Our interest was in processes that affect gender differences in alcohol use with a focus on family variables, namely parental monitoring, parental involvement in adolescent school performance, and parental empathy. It seems unlikely that the impact of such fundamentals on alcohol use would notably shift over 15 years, especially given these variables have a long and successful history in the alcohol literature more generally [17]. Attitudes change over time, but the core family mechanisms that impact that change likely continue from one generation to the next, i.e., protective parenting likely maintains impact across generations. At the very least, the current study established a baseline for comparisons with future research.

The current study had notable strengths as well as limitations. First, the study used large representative samples of early adolescents in four major cities in China, which increases the generalizability of its findings. Second, the study was comprehensive in that it studied four distinct drinking outcomes and found similar trends with respect to each outcome. Third, the study was innovative in exploring non-linearity in outcomes as a function of the (ordinally scaled) parenting dimensions. This method allowed us to identify important trends in the data that would have been masked by applying more traditional linear modeling. Fourth, our study focuses on adolescents’ perceived parenting behavior rather than parenting behavior per se. Parents’ self-reported behavior is less predictive than adolescents’ perceptions of parenting in relation to adolescent risk behaviors including alcohol use [29–31]. More comprehensive research can collect data from both parents and adolescents to fully study the differences between parent and adolescent reported parenting behaviors when predicting Chinese adolescent drinking. Fifth, we tested for estimated effects of parenting attributions holding other parenting attributions constant, giving us a more nuanced appreciation of the unique variance of each attribution in a multivariate context.

Among the limitations, the study did not include rural youth, so its results can only be generalized to urban youth. Second, given the nature of the assessment environment, the questions surrounding attributions of parental behaviors were somewhat limited by using single-item measures. Nevertheless, the parenting measures were predictive in theoretically coherent ways, which provides reassurance for the validity of the measures. Third, the study relied on adolescents’ self-reports of their alcohol use behavior, which also suggests caution in result interpretation. Self-reports, for example, can be subject to self-presentation bias. To minimize this, the survey was anonymous, voluntary, and was accompanied by instructional sets that emphasized the importance of truthful responding. Although we doubt there was systematic bias, it can’t be ruled out definitively. Fourth, the modeling is subject to omitted variable bias and must be interpreted with caution. For example, parental alcohol use could influence both adolescent drinking and adolescent perceptions of perceived parenting behavior [59]. Parent alcohol use could not be reliably measured given this was a student survey of young adolescents. We doubt, however, that parental alcohol use could account for the complex pattern of results we observed between parenting and alcohol use, nor would we expect there to be notable differences in parental drinking as a function of the gender of their child. Finally, the research was cross-sectional, which weakens confidence in any causal inferences. Despite these limitations, the research provides numerous interesting results and leads for more detailed follow-up in future research.

## Conclusions

To conclude, Chinese male adolescents exhibited higher levels of drinking frequency, drinking amount, heavy drinking, and problems associated with drinking than female adolescents. Gender differences in these drinking outcomes were non-linearly and negatively associated with perceived parental monitoring, parental involvement in adolescent school performance, and parental empathy. The reduced gender differences were mainly due to lower levels of drinking for male adolescents being associated with more positive parenting attributions rather than for female adolescents. For the latter, the associations were negligible. Parent-based alcohol use prevention and intervention programs should take gender differences into account.

## Data Availability

The datasets analyzed during the current study are publicly available in World Health Organization Global School-Based Student Health Survey (GSHS), http://www.who.int/chp/gshs/en/.

## Abbreviations

GSHS: Global School-Based Student Health Survey
